# A tree-based approach to identify indispensable foods in minimum-cost food baskets

**DOI:** 10.3389/fnut.2024.1425749

**Published:** 2024-09-18

**Authors:** Melissa F. Koenen, Romée Geelen, Marleen Balvert, Hein Fleuren

**Affiliations:** ^1^Department of Econometrics and Operations Research, Tilburg School of Economics and Management, Tilburg University, Tilburg, Netherlands; ^2^Zero Hunger Lab, Tilburg School of Economics and Management, Tilburg University, Tilburg, Netherlands

**Keywords:** food and nutrition security, cost resilience, diet optimization, food baskets, linear programming

## Abstract

**Introduction:**

Optimization techniques, such as linear programming, can be used to estimate the minimum cost of a nutrient-adequate food basket, to determine if individuals or households can afford nutritious diets. These cost estimates typically account for seasonal fluctuations but often overlook significant disruptions in the availability of affordable nutritious food that can severely impact food and nutrition security.

**Methods:**

This paper proposes a tree-based method, the binary search tree, to assess the resilience of the cost estimate of the minimum-cost food basket. In particular, this method aims to identify indispensable foods in these baskets — those whose unavailability would lead to a substantial cost increase. The binary search tree operates by iteratively excluding essential food items while ensuring the construction of minimum-cost nutritious baskets. It considers all relevant combinations of foods up to a specified size and avoids unnecessary optimizations, thereby saving computation time. We describe how the resulting tree can be evaluated and condensed to capture only the necessary information for decision makers. The construction and evaluation of the binary search tree are independent of the (dietary) restrictions or type of optimization model (i.e., linear, non-linear or integer) included.

**Results:**

In general, the binary search tree can identify all (combinations of) foods whose exclusion leads to a significant cost increase of a nutritious food basket. Furthermore, it can detect possible substitute effects between foods and identify key limiting nutrients. A case study is presented in which the binary search tree is applied to data from Ebonyi, Nigeria, modeled using linear programming. We report all combinations of up to five foods that, when unavailable, can impact food and nutrition security in Ebonyi.

**Conclusion:**

The BST can provide insights into local food and nutrition security when facing drastic disruptions in access to nutritious foods by identifying indispensable foods. Its results can be used to inform and design interventions in the context of humanitarian operations.

## 1 Introduction

As defined at the Food and Agriculture Organization World Food Summit in 1996 (1, 2), food security encompasses the physical availability of food, the economic and physical access to food, food utilization, and the stability of these former factors over time. Since the onset of COVID-19, global food security has declined and seems on a further decline because of conflict and climate change (3). The number of people affected by hunger has increased by more than 150 million from 2019 to 2021, resulting in at least 702 million people in hunger (3). Moreover, as dietary costs increase with the dietary quality and diversity, healthy diets are often unviable for low-income households (4).

Operations research techniques are nowadays commonly used to aid humanitarian organizations in the battle against hunger and to improve food security (5–7). The United Nations (UN) World Food Programme for example uses diet optimization to estimate the cost of a nutrient-adequate food basket for a household within a certain region (5, 7, 8), as part of the Fill the Nutrient Gap (FNG) analyses (8, 9). This cost does not necessarily reflect the actual cost of a healthy and palatable diet, however, it provides insights into the minimum amount of food expenditure that is required to afford a nutritious diet. In case this amount of required expenditure is not within a reasonable ratio of the household income, policy interventions [e.g., improving school lunches (10)] are proposed to improve local food security and the nutritional value of diets.

The FNG is a collaborative analytical process conducted in consultation with stakeholders such as government, academia, United Nations, donors, private sector and civil society (8). Where data is deemed inadequate or unavailable, one of the steps of the FNG is to perform a market analysis where the local prices of an extensive list of available food items are gathered and validated. This is usually done at multiple points in time to account for seasonal fluctuations in crop yield and therefore in price levels. The cost estimate of a nutritious diet can differ significantly throughout the year and this is taken into consideration when proposing interventions.

Although the proposed food baskets take seasonal fluctuations into account, they do not consider severe incidental disturbances on the supply side. Conflict, climate change, epidemics, economic and political stability, and supply chain disruptions impact access to affordable nutritious meals. These factors can either disrupt access to a specific food item (bacteria affecting olive trees (11) or fungi affecting bananas (12)), to various food items from a specific geographical location (war in Ukraine impacting grain harvest and export (13), heavy hailstorms and sudden cold in Morocco and Spain impacting tomato and pepper harvest (14), or bird flu resulting in the dispatching of chickens in Japan (15)), or to a mixture of arbitrary food items (vessel obstructing the Suez Canal (16)).

In this paper, we propose a method to investigate how resilient the current cost estimate of the most affordable nutrient-adequate food basket is when facing drastic disturbances. In particular, we are interested in finding those food items that would lead to a large cost increase if they become unavailable. Techniques that generate nearly optimal solutions, such as the hop-skip-jump (17), could be used to investigate whether other nutritious food basket compositions are possible within a certain price range. The downside of these techniques is that it is difficult to pinpoint which (combinations of) food items are essential to ensure the affordability of a minimum-cost nutritious food basket. That is, these techniques are designed to construct solutions that are as different as possible from previously found solutions, rather than identifying which (combinations of) items are relevant for the cost. Instead, to be able to perform this analysis we present a binary search tree (BST) that iteratively carries out a cost minimization while excluding food items that are relevant to construct a minimum-cost nutritious basket. The BST shows which (combinations of) food items are essential to construct a minimum-cost nutritious food basket by comparing the cost of the different food baskets within the tree. Moreover, the BST can be used to derive possible substitute effects between food items and to identify which nutrient requirements are relatively easy and difficult to meet. The main advantage of this procedure is that it is intuitive and easy to incorporate within an existing diet optimization structure.

## 2 Methodology

The general idea behind the BST is that it models unavailability by excluding food items that are in the currently constructed basket one by one. By assessing which (combinations of) food items are excluded and how they influence the cost of the newly constructed baskets, it is possible to determine which (combinations of) food items are essential to maintain a similar nutritional value. One way to construct these food baskets within the BST is by using the optimization technique linear programming (LP), which finds the minimum-cost food basket given certain nutritional requirements while taking cultural habits into account. Note that we do not need any assumptions on the restrictions and the type of optimization model (i.e., continuous or integer food items) to construct and evaluate the BST.

We first explain how to construct the complete BST in Section 2.1 and we describe how the tree can be interpreted in Section 2.2 by only deriving relevant information. For simplicity the explanation is based on a toy example—a small example to demonstrate our method—for a household with one individual, however its principles can be readily applied to larger households. Then, in Section 2.3 we present a case study for a household of five in the region Ebonyi in Nigeria, where we explain the underlying LP model that is used to build the food baskets within the BST. Here, we make similar assumptions as in Cost of the Diet (5), a diet optimization model developed by Save the Children and used by the UN World Food Programme, among others.

### 2.1 Construction of the BST

**Figure 1:**
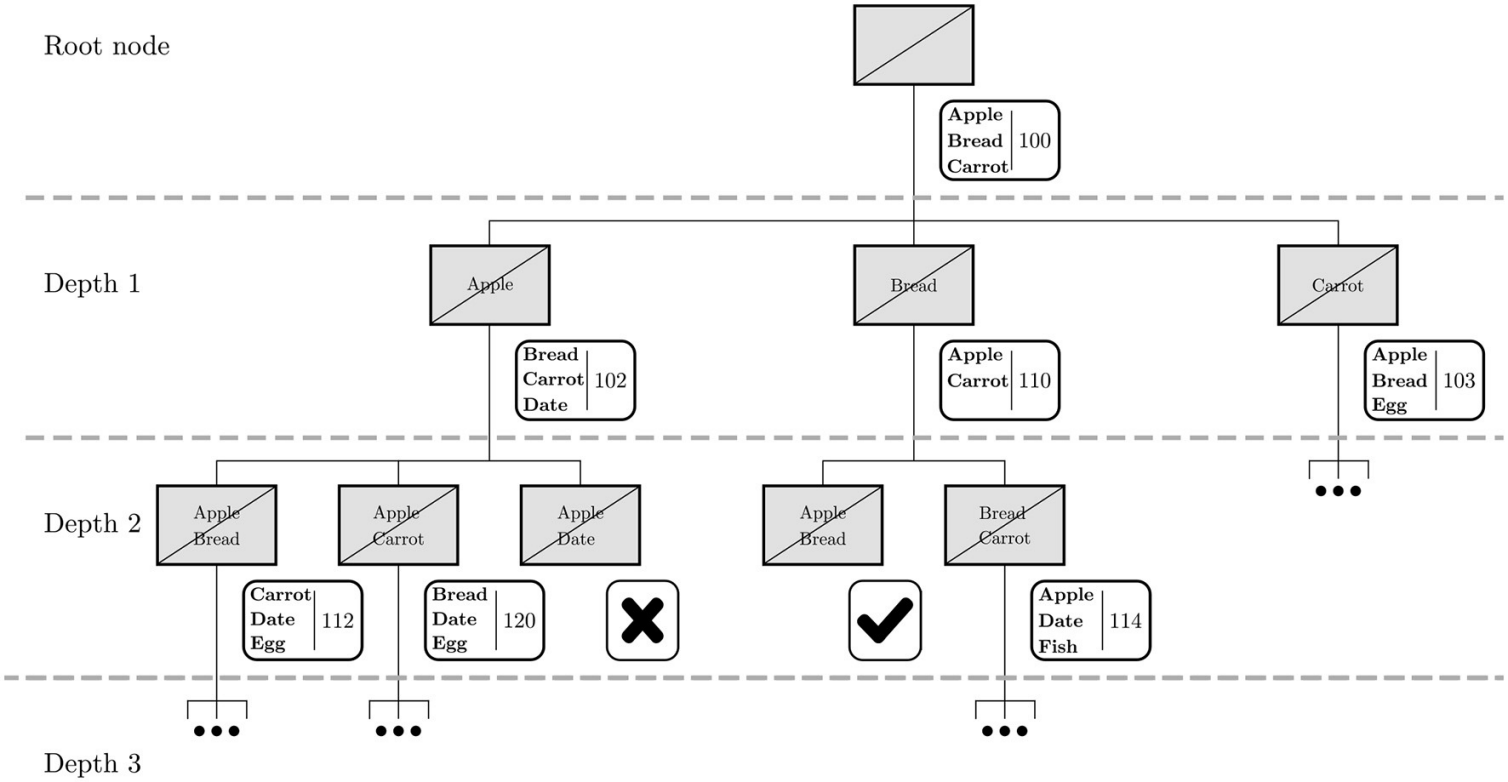
Example of a BST for a small toy example. The gray boxes show which food items are excluded, and the white boxes show the food items that are in the corresponding optimal food basket and its respective cost. In case it is not possible to construct a food basket that meets all dietary restrictions, this is indicated with a cross. In case a specific combination of excluded food items, or exclusion set, has already been considered in the tree, this is indicated with a check mark.

We explain the BST through a toy example for a single individual, shown in Figure 1. We assume in the remainder of the text that each of the solutions are found using a minimum-cost optimization with context-specific restrictions (e.g. nutritional requirements, cultural preferences, dietary guidelines) that hold for all solutions.

The BST starts with an initial solution, the root node, where there are no restrictions on the availability of the food items. The resulting food basket contains a variety of food items, which in this toy example consists of apple, bread and carrot with a cost of 100, see Figure 1. For each of these items a new node is created, where this specific item is excluded. This takes place at depth 1 of the tree. As each of these items is removed in their particular branches, the initial solution is not feasible anymore and therefore new food baskets have to be constructed. As this problem is more restricted, the cost of the food basket can never decrease. Within our example this results in three different food baskets with a cost of 102, 110, and 103, respectively.

The newly constructed baskets usually contain a few additional items that may serve as a substitute for the excluded food item. For example, when only excluding apple, date is included in the food basket instead. For each of the items of the newly constructed basket, a new node is created excluding the corresponding item. This takes place at depth 2 of the tree. This procedure can be repeated to create a tree of arbitrary depth, showing the effects on the food basket when excluding various combinations of food items. We refer to the exclusion of a particular combination of food items as an exclusion set.

While constructing the tree, a specific exclusion set might be encountered again. For example, in depth 2 of the tree the combination apple and bread is found twice: once where in the parent node only apple was excluded, and once where in the parent node only bread was excluded. In that case, one of the branches can be pruned from the tree, i.e., this solution is not considered any further. This saves computation time and avoids unnecessary duplicates. A node may also be pruned when not enough food items are left to fulfill the dietary restrictions. In our example, this happens when apple and date are both unavailable.

The number of exclusion sets can increase exponentially when traversing too deeply in the tree. This affects the computation time, as many more optimizations need to be performed. To avoid an unnecessarily complex tree, the tree is grown until a certain depth is reached. This is a reasonable approach, as it is unlikely that many food items will be unavailable at the same time except in the event of a large disaster. The way the BST is constructed ensures that all combinations up to a certain number of items are found that result in significant cost increases or infeasible solutions.

### 2.2 Evaluation of the BST

After the BST has been constructed, it can be used to evaluate which (combinations of) food items are essential to construct minimum-cost nutrient-adequate food baskets. Furthermore, the BST can be used to detect possible substitution effects between (combinations of) food items. Here, we refer again to our toy example to explain how this can be derived.

To identify essential (combinations of) food items, we try to find exclusion sets that either result in infeasible solutions or in major cost increases. The clearest indicator of an essential combination is in the occurrence of an infeasible solution. This means that without these food items, it is impossible to construct a food basket that adheres to all requirements. In practice, this only happens in very deep layers of the tree as usually enough food items are available that can fulfill the (nutritional) requirements. From our toy example, we conclude that the combination of apple and date is essential.

In the other case, we define a major increase as a cost increase of more than *x%*, where the threshold can be defined based on the local context. Given an exclusion set that resulted in a major cost increase, we want to investigate whether this increase is actually caused by this specific combination of items, or can be explained by the exclusion of a subset^1^ of those items. For example, if excluding item A results in a significant cost increase, then any exclusion sets that contain item A will result in at least the same cost increase. If the cost increase of excluding items A and B is comparable to just excluding item A, then we cannot attribute the cost increase to that combination. We conclude that only excluding item A in itself is a risk. However, if the cost increase of excluding items A and B is much higher than to just excluding item A, then we can attribute it to that combination. We conclude that on top of the risk of excluding item A, is the risk of excluding items A and B together.

In this case, we determine which (combinations of) food items are essential, by considering the price increase of a food basket relative to the highest cost of other food baskets that omit a subset of the exclusion set. We refer to this as a subset cost increase. We elaborate on this concept by showing some examples, where for illustration purposes we assume that exclusion sets that increase the subset cost by more than 5% are regarded as a potential risk to food and nutrition security.

In our first example we have a food item that is essential, but excluding the item together with another item is not an additional risk. In depth 1, only bread is considered to be an essential food item, as excluding it increases the price from 100 to 110, see Figure 1. In the left branch of depth 2, excluding both apple and bread results in a price of 112. Compared to the cost corresponding to the parent node “excluding apple”, which was 102, this may indicate that although apple is not an essential food item on its own, excluding both bread and apple does lead to a major cost increase from 102 to 112. However, this cost increase is not attributed to the combination apple and bread, but mainly to the exclusion of bread. Recall that excluding bread led to a cost of 110, meaning that additionally excluding apple leads to a cost increase of only 1.8%. Thus, we conclude that the exclusion of apple and bread together is not a potential risk, as the subset cost increase is 1.8%.

A combination that can be seen as a risk is apple and carrot. Only excluding apple or carrot is not that problematic as the cost would rise to 102 and 103, respectively. However, excluding them both raises the cost to 120. Hence, excluding apple and carrot simultaneously leads to an increase of 16.5% w.r.t. 103, where only carrot was excluded. The subset cost increase in this case is thus 16.5%.

Similarly, we can determine for larger combinations whether they are essential. For example, if one wants to know whether the exclusion set apple, bread, carrot in depth 3 would be a problem, one has to consider the price increase relative to the highest cost of the three baskets that exclude (i) apple and bread, (ii) apple and carrot, and (iii) bread and carrot. Here, we compare based on the cost of excluding (ii), 120, as the resulting food basket is the most expensive one among the three.

Besides finding which food items are relevant for maintaining more affordable and nutritious food baskets, the BST can be used to evaluate possible substitute effects. We only regard food items as a relevant substitute for each other, when there are no other affordable foods available that can fulfill their role. If a food item is unavailable, then the most affordable substitute will be present in the newly constructed basket. However, if both a food item and its substitute are not present, and there are no other affordable substitutes, then it is either not possible to construct a basket or the cost of the basket will increase considerably. Hence, substitutes can be identified by looking at the combinations of food items that, when excluded together, either lead to an infeasibility or to a substantial cost increase relative to excluding the items individually. Again, an infeasibility is easy to detect, and in our toy example, it holds that apple and date are considered substitutes for each other.

In the case of a substantial cost increase, it is expected that when excluded together the increase in cost will be much higher than the sum of the increases in cost of excluding the foods separately. To investigate whether (combinations of) food items are substitutes, we introduce the concept of marginal cost increase. In short, the marginal cost increase of a food item (or combination of) is the expected cost increase (%) when excluding it relative to the root node basket. In the next few paragraphs, we define the marginal cost increase more explicitly and provide some examples to clarify the concept.

To determine the marginal cost increase, we consider all possible partitions of the exclusion set,^2^ where a partition is a way to divide a set of items into multiple sets of items such that each item is only in one set. We sum for each subset in the partition their relative cost increase with respect to the root node cost. The highest sum is the marginal cost increase. If the % cost increase of the exclusion set, which is relative to the root, is much higher than the determined marginal cost increase, we suspect a substitute effect.

For our toy example, it holds that apple and bread are no substitutes for each other. Only excluding apple results in a 2% increase with respect to the root node, and only excluding bread results in a 10% increase. This is a 12% marginal increase, which matches with the optimized cost increase. Similarly, bread and carrot are not substitutes for each other, as 10%+3% = 13% does not differ substantially from 14% increase. Apple and carrot, however, seem to have a substitute effect. The sum of their marginal cost increases is 2%+3% = 5% which is much smaller than the optimized 20%.

Although our toy example does not illustrate depth 3, we will describe how to determine the marginal cost increase of an exclusion set of size 3. If we consider the exclusion set apple, bread and carrot in depth 3, then we need to consider four possible partitions: (i) {apple}, {bread}, {carrot}, (ii) {apple}, {bread and carrot}, (iii) {apple and bread} and {carrot}, and (iv) {apple and carrot} and {bread}. Note that all items are included in each of the partitions. Based on our tree the marginal costs for each of the partitions are i) 2+10+3 = 15, ii) 2+14 = 16, iii) 12+3 = 15, and iv) 20+10 = 30. Thus, the marginal cost increase of apple, bread and carrot is 30%. Depending on the optimized cost of excluding apple, bread and carrot, we can state whether there is a substitute effect between {apple and carrot} and {bread}.

Based on the BST of the toy example, we can now conclude that as a single food item, bread is relevant for constructing minimum-cost food baskets as excluding it increases the price by 10%. For food item pairs, we can conclude that the exclusion of apple and carrot together is expensive, as it increases the total price to 120. This is a 16.5% increase compared to only excluding carrot. We can also conclude that the exclusion of apple and date together is especially alarming as in that case no nutritious diet can be constructed. Furthermore, we find that apple and carrot, and apple and date have a substitute effect.

Note that the conclusions of the BST analysis depend on the available foods, their nutritional content, and their prices. This implies that the identified indispensable foods and substitute effects are both time- and region-specific.

### 2.3 Case study

In this section we show how the BST can be used to investigate the cost sensitivity of food baskets w.r.t. availability of food items. We do so by presenting a case study for the Ebonyi region in Nigeria for a household of five. In Section 2.3.1 we first explain the used diet optimization model for which the same assumptions are made as for the Cost of the Diet model (5, 18). Then, in Section 2.3.2 we describe the accompanying data set for the Ebonyi region, which was shared by the UN World Food Programme.

#### 2.3.1 Optimization model

We adopt the optimization model as proposed in (18), where we apply some simplifications to the notation. This model uses LP optimization to find the most affordable food basket while adhering to nutritional requirements and cultural habits of a single individual. We refer to (19) and (20) for more information regarding general LP optimization. Note that depending on the context, the user can set additional/different constraints (e.g. additional nutrients, ratios between nutrients, palatability constraints, integer intake of foods) or specific values for the parameters (e.g. more restrictive intake of certain nutrients). This does not alter the construction and evaluation process of the BST.

Let *x*_*i*_ denote the decision variable that states the amount of food item i∈I in edible grams included in the food basket, where I represents the set of locally available food items. Furthermore, let N be the set of micro- and macronutrients. Let G represent the set of all food groups (e.g. grains or fruits) and $I_{g}$ denote the set of food items belonging to food group g∈G. Then, the corresponding diet optimization problem for a single individual can be described as:


(1) $$\begin{array}{ll} \text{(CotD)}=\min & \underset{i\in I}{\sum }c_{i}x_{i} \end{array}$$

(2) $$\text{ s.t. }\sum_{i\in ℐ}a_{i,n}x_{i}\geq {\underline{b}}_{n}\;\forall n\in N$$

(3) $$\begin{array}{ll} \;\underset{i\in I}{\sum }a_{i,n}x_{i}\leq {\bar{b}}_{n} & \;\forall n\in N \end{array}$$

(4) $$\;\frac{x_{i}}{p_{i}}\geq \underline{f}\;\forall i\in ℐ$$

(5) $$\begin{array}{ll} \;\frac{x_{i}}{p_{i}}\leq \bar{f} & \;\forall i\in I \end{array}$$

(6) $$\begin{array}{ll} \;\underset{i\in I_{g}}{\sum }\frac{x_{i}}{p_{i}}\leq s_{g} & \;\forall g\in G \end{array}$$

(7) $$\begin{array}{l} \;\underset{i\in I}{\sum }x_{i}\leq w. \end{array}$$

Objective (1) minimizes the cost of the food basket, where *c*_*i*_ denotes the cost per edible gram of food item i∈I. Constraints (2) and (3) ensure nutritional feasibility by restricting the minimum and maximum intake of nutrients, respectively. Here, *a*_*i, n*_ is the amount of nutrient n∈N per gram of food item i∈I and *b*_*n*_ and ${\bar{b}}_{n}$ represent the minimum and maximum allowed intake of nutrient n∈N, respectively. Constraints (4) and (5) impose a minimum and maximum intake on each food item, where *p*_*i*_ denotes the portion size for food item i∈I and *f* and $\bar{f}$ represent the minimum and maximum number of servings, respectively. Constraints (6) promote diversity within the food basket by not including too many items from the same food group, where *s*_*g*_ is the maximum number of servings of items from food group g∈G. Constraint (7) limits the total food weight, *w*, of the food basket. Here, we implicitly assume that *p*_*i*_>0 and $\underline{f},\bar{f}\geq 0$ to ensure that *x*_*i*_≥0.

The model above is used to construct food baskets for individuals. We now briefly explain how it can be used to construct a BST for a whole household. When constructing a node within the household BST, we determine for each individual their optimal basket using (CotD) given the exclusion set. We aggregate the individual's baskets into one basket and use that composed basket to select the items that will be excluded in the next depth of the household BST. Note that it is only necessary to re-optimize an individual's basket when the newly excluded food item is within the individual's food basket of the parent node. In case the food item is not in the basket composition of the parent node, the composed food basket and the cost of this individual can be passed on to the child node. Evaluation of the BST remains the same as explained in Section 2.2.

#### 2.3.2 Data

For testing our approach, we consider a typical household used within the FNG analyses of the UN World Food Programme (8, 9), consisting of a 1-year-old breastfed child, a school-age child, an adolescent female, a lactating female and an adult male. In Supplementary material Section 1 we report the daily nutritional requirements of these five household members for all considered nutrients N and state the maximum food weight *w* for each individual. Here, we assume that the breastfed child receives a daily amount of 532 grams of breastmilk (18), and we take this into account for the reported nutritional requirements and maximum food weight. Supplementary material Section 1 also lists the available food items and the corresponding food groups, obtained for the Ebonyi region, Nigeria. Furthermore, it reports for each household member their portion size *p*_*i*_ of each food item. The cost and nutritional data corresponding to these food items are reported in (7), and were gathered by the UN World Food Programme Nigeria country office. The cost data were gathered and validated as part of the 2022 FNG analysis in Nigeria (21). Throughout our analysis, we assume *f* = 0, $\bar{f}=3$, and *s*_*g*_ = 15 for all g∈G, in accordance with the approach of the FNG analyses (5).

## 3 Results

In this section, we present the results of the BST for the Ebonyi case study where we grow the BST to a depth of five and we show which (combinations of) food items are essential to keep the minimum-cost food basket as affordable as possible while adhering to nutritional and cultural requirements. Here, we consider all (combinations of) food items with a subset cost increase of at least 5%. All experiments are performed on an HP ZBook Studio G4 operating on Windows with Intel Core i7-7700HQ, running on a 2.80GHz processor with a memory of 16 GB RAM, using Gurobi 10.0.0 as the LP solver.

First, we need the composition of the minimum-cost basket constructed with (CotD) as a baseline. Table 1 shows the daily minimum-cost food basket for the household which is determined by totaling the constructed food baskets of each of the individuals in the household, along with its total cost. Recall that this food basket is a hypothetical diet that indicates the minimum cost required to afford a nutritious diet, rather than proposing an actual consumable diet.

**Table 1:** Daily composition in grams of the minimum-cost food basket for each of the individuals in the Ebonyi household, and the total daily composition in grams and total daily cost in NGN.

	**Breastfed child**	**School-age child**	**Adolescent female**	**Lactating female**	**Male**	**Total**
Groundnut, shelled, dried, raw	6.8	66.6	101.9	79.8	72.8	327.9
Lamb, liver, raw	0.4	2.7	20.6	83.4	2.7	109.8
Leaf, amaranth, raw	84.4	130.6	426.4	212.5	220.2	1074.1
Millet, pearl, whole grain, raw	60.5	17.7	134.6	612.4	233.0	1,058.1
Sorghum, whole grain, raw	78.0	290.0	342.1	0.0	404.5	1,114.6
				Cost		1,249.79

This food basket adheres to the provided (nutritional) requirements.

For the minimum-cost food basket, fat and pantothenic acid are at their lower limit for each individual, and calcium is at its lower limit for everyone except the adolescent female. In addition, vitamin B12 is at the lower limit for the breastfed child and the male, iron absorbed is at the lower limit for the adolescent female, and vitamin A is at the lower limit for the school-age child and at the upper limit for the adolescent female. We refer to Supplementary material Section 2 for an overview of the nutritional content of the minimum-cost food basket.

In depth 1 of the BST we exclude each of the food items in Table 1 individually. For our results we allow the tree to grow to a depth of five, which implies that at most five items are simultaneously unavailable. As explained in Section 2.1, the BST only excludes food items that were included in the food baskets of the direct parent.

The resulting BST is formed in 185 seconds and consists of 1425 nodes, however not all exclusion sets are relevant. As in line with the example in Section 2.2, we only consider exclusion sets that would imply a cost increase of at least 5% compared to food baskets constructed using any of its subsets, referred as the subset cost increase.

Table 2 shows all exclusion sets that lead to a relatively high increase in subset cost of at least 5%. For example, when only single items are excluded the exclusion of “lamb, liver” results in an increase in subset cost of 11.6%. The resulting food basket does not contain “lamb, liver” anymore, but different items such as “fish, dried”. We conclude that the availability of “lamb, liver” is relevant for the affordability of the minimum-cost nutritious food basket. Similarly, in the situation where two items are not available at the same time, the exclusion of “lamb, liver” and “fish, dried” results in a subset cost increase of 21.3% compared to just excluding “lamb, liver”, its most expensive subset. This shows that the unavailability of ‘fish, dried' in itself does not cause any problems, while “fish, dried” is important when “lamb, liver” is unavailable as well.

**Table 2:** Overview of (combinations of) food items that when excluded cause a relatively high increase in cost for the Ebonyi case study.

					**Cost of new basket (NGN/day)**	***%* Cost increase w.r.t. root node**	***%* Marginal cost increase w.r.t. root node**	***%* Cost increase w.r.t. all subsets**
**Zero items excluded**								
—					1,249.79	0.0	0.0	0.0
**One item excluded**								
Lamb, liver					1,394.67	11.6	11.6	11.6
**Two items excluded**								
Sorghum	Lamb, liver				1,467.25	17.4	16.3	5.2
Leaf, amaranth	Sorghum				1,382.65	10.6	9.1	5.7
Leaf, amaranth	Sesame seeds				1,401.56	12.1	4.4	7.4
Leaf, amaranth	Lamb, liver				1,523.99	21.9	16.0	9.3
Millet	Sorghum				1,460.32	16.8	6.2	11.6
Lamb, liver	Fish, dried				1,691.47	35.3	11.6	21.3
**Three items excluded**								
Leaf, amaranth	Lamb, liver	Leaf, eggplant			1,614.63	29.2	21.9	5.9
Leaf, amaranth	Lamb, liver	Fish, dried			1,793.93	43.5	39.8	6.1
Leaf, amaranth	Sesame seeds	Leaf, eggplant			1,498.75	19.9	12.1	6.9
Millet	Sorghum	Lamb, liver			1,578.10	26.3	28.4	7.6
Leaf, amaranth	Millet	Lamb, liver			1,639.65	31.2	23.5	7.6
Lamb, liver	Fish, dried	Fish, mackerel			1,949.25	56.0	35.3	15.2
**Four items excluded**								
Leaf, amaranth	Millet	Lamb, liver	Fish, dried		1,899.72	52.0	45.1	5.9
Leaf, amaranth	Lamb, liver	Oil, palm	Leaf, eggplant		1,710.79	36.9	29.2	6.0
Leaf, amaranth	Millet	Sorghum	Lamb, liver		1,753.06	40.3	38.8	6.9
Leaf, amaranth	Lamb, liver	Sesame seeds	Leaf, eggplant		1,728.26	38.3	31.5	7.0
Leaf, amaranth	Millet	Lamb, liver	Leaf, eggplant		1,757.15	40.6	31.2	7.2
Millet	Sorghum	Lamb, liver	Fish, dried		1,889.29	51.2	52.2	7.8
Leaf, amaranth	Sesame seeds	Soybean	Leaf, eggplant		1,637.05	31.0	19.9	9.2
Leaf, amaranth	Lamb, liver	Leaf, eggplant	Egg, chicken		1,792.06	43.4	29.2	11.0
Lamb, liver	Fish, dried	Fish, mackerel	Egg, chicken		2,581.01	106.5	56.0	32.4
**Five items excluded**								
Leaf, amaranth	Sorghum	Cucumber	Rice, white	Mushroom	1,502.40	20.2	15.7	5.1
Leaf, amaranth	Millet	Sorghum	Lamb, liver	Fish, dried	2,000.25	60.0	60.4	5.3
Leaf, amaranth	Millet	Sorghum	Sesame seeds	Soybean	1,608.18	28.7	29.2	5.3
Leaf, amaranth	Millet	Lamb, liver	Oil, palm	Leaf, eggplant	1,859.69	48.8	40.6	5.8
Leaf, amaranth	Lamb, liver	Sesame seeds	Oil, palm	Leaf, eggplant	1,830.28	46.4	38.3	5.9
Leaf, amaranth	Lamb, liver	Sesame seeds	Leaf, eggplant	Egg, chicken	1,907.59	52.6	43.4	6.4
Leaf, amaranth	Millet	Sesame seeds	Soybean	Cowpea	1,578.71	26.3	18.3	6.8
Sorghum	Lamb, liver	Rice, white	Mushroom	Cucumber	1,594.80	27.6	22.8	7.0
Leaf, amaranth	Millet	Sorghum	Soybean	Cowpea	1,665.08	33.2	24.6	8.4
Millet	Sorghum	Lamb, liver	Fish, dried	Fish, mackerel	2,201.80	76.2	72.8	8.8
Leaf, amaranth	Millet	Lamb, liver	Leaf, eggplant	Egg, chicken	1,968.81	57.5	44.9	9.9
Leaf, amaranth	Lamb, liver	Fish, dried	Leaf, eggplant	Egg, chicken	2,134.77	70.8	49.5	14.3
Leaf, amaranth	Lamb, liver	Fish, dried	Sesame seeds	Leaf, eggplant	2,142.90	71.5	55.3	14.7
Leaf, amaranth	Lamb, liver	Oil, palm	Leaf, eggplant	Carrot	2,335.87	86.9	36.9	36.5
Leaf, amaranth	Sesame seeds	Soybean	Leaf, eggplant	Fish, dried	2,957.86	136.7	31.0	80.7

The information is extracted from analyzing the BST. The overview is sorted on the number of excluded items and the % cost increase w.r.t. subsets. For readability, the names of the food items are shortened. All food baskets adhere to the provided (nutritional) requirements.

“Lamb, liver” and “leaf, amaranth” are present in many of the exclusion sets that have a large increase in cost. Although only excluding “leaf, amaranth” does not increase the subset cost by more than5%,^3^ the inclusion of “leaf, amaranth” is relevant to keep the overall food basket more affordable when other affordable nutritious foods are not available. This indicates that “leaf, amaranth” is dispensable and can easily be replaced by combinations of other food items unless some of these other food items are unavailable as well.

Table 2 reports the % cost increase relative to the minimum-cost food basket, referred to as a root node cost increase, and it reports the marginal % cost increase. The latter is used to detect whether foods may have a substitute effect that is more expensive to replace with other foods, as explained in Section 2.2. A substitute effect might be present when the difference between the root node cost increase and the marginal cost increase is large. Note that this difference often has a positive correlation with the subset cost increase, however, a relatively high subset cost increase does not guarantee a large difference (see e.g. the exclusion set “sorghum” with “lamb, liver”).

An example of a substitution effect is when excluding “millet” and “sorghum” simultaneously. The marginal increase is determined by a 1.6% increase in root node cost of excluding “millet” and 4.7% when excluding “sorghum”. Note that both outcomes are lower than 5% and not reported in the above table. If these food items are no substitutes for each other, then one would expect the marginal cost to increase by 6.2%.^4^ The increase in root node cost, however, is 16.8% which is a significant 10.6 percentage points difference. This implies that removing them individually forms a basket where the other item is used as a relevant substitute, and removing them simultaneously results in a high increase in cost as there is no other food available with similar nutrients for that cost.

An example where no relevant substitution effect seems present is the exclusion of “leaf, amaranth”, “lamb, liver” and “fish, dried”. Here, the marginal increase is determined by summing 35.3% of the combination “lamb, liver” and “fish, dried” together with 4.4% of “leaf, amaranth”. The increase in root node cost is 39.8%,^5^ which is only a 3.8 percentage points difference. Here, “leaf, amaranth” does not act as a prominent substitute for the combination “lamb, liver” and “fish, dried”, and vice versa, as otherwise the cost would have increased more.

A prominent substitution effect happens when excluding animal-source foods as they are the only source of vitamin B12. This can best be seen by traversing from the largest exclusion sets to the smallest. Let us consider the exclusion of “lamb, liver”, “fish, dried”, “fish, mackerel” and “egg, chicken”. This exclusion results in a 32.4% subset cost increase and has a large difference of 50.5 percentage points between the marginal and root node cost increase. When looking at the three items excluded it is apparent that this increase in cost is caused by excluding “egg, chicken” on top of “lamb, liver”, “fish, dried” and “fish, mackerel”. Thus, “egg, chicken” is a relevant substitute for the combination “lamb, liver”, “fish, dried” and “fish, mackerel” in that particular basket. Similarly, when looking at two items excluded “fish, mackerel” is a relevant substitute for “lamb, liver” and “fish, dried”, as there is a large difference between the marginal and root node cost increase. This reasoning can be extended up to the exclusion of “lamb, liver” where “fish, dried” is a relevant substitute for “lamb, liver”.

Thus, the above condensed table can be used to determine which (combinations of) food items are essential to construct minimum-cost nutritious food baskets. Moreover, it shows possible substitutes for foods when the marginal cost increase is significantly higher than the root node cost increase.

Besides that, the constructed BST can also help to identify nutrient requirements that are relatively difficult and easy to meet, given the available food items. Using the food basket composition of all exclusion sets, we can determine the nutrients that often approach their lower or upper limit. Our case study shows that the requirements for protein, iron, magnesium, vitamin B1, vitamin B6 and zinc are easily met. In most cases, the niacin requirement does not seem a problem. The nutrients that are usually on their lower limit are fat, calcium, pantothenic acid and vitamin B12. Furthermore, iron absorbed is usually at the lower limit for the breastfed child, adolescent female and lactating female. As an example, Supplementary material Section 3 provides the nutrient output for each of the food baskets of Table 2 for the adolescent female.

## 4 Discussion

In this discussion, we first summarize the core principle of the BST before discussing its main assumptions. Then, we recap the key insights of the BST analysis for the Nigeria case study. We finish this section by explaining how the results of a BST analysis could benefit the current operations of the UN World Food Programme, and we suggest some additional applications of the BST.

### 4.1 Summary of the BST

This paper presents a tree-based method, the BST, that identifies indispensable foods in a minimum-cost nutrient-adequate food basket. The method only considers the exclusion of relevant combinations of foods, i.e. foods that when excluded would increase the cost, which saves computation time. By considering only combinations that would significantly increase the cost, the resulting output can be condensed in a single table containing only relevant information. The output helps to find possible substitutes between foods, and the output of the BST is able to identify nutrients that are overall easy or difficult to meet given the available foods.

We have suggested two measures that can be used to evaluate the BST: the subset cost increase and the marginal cost increase. Although the two measures are slightly related, they each fulfill a different role in the analysis. The former is used to determine which food combinations can pose a risk to the affordability of nutritious diets when unavailable, and the latter is used to investigate possible substitute effects between food items. The results of the two could even be combined to further reduce the size of the overview table. That is, to only show (or to highlight) the combinations that, besides a high subset cost increase, have a large difference in the root node cost and marginal cost as well. This would then only present the combinations of which the cost increase was not expected.

To illustrate the BST we made specific assumptions for the example and the case study. That is, we considered a maximum depth of five for the BST, and we only reported the subsets of foods that had a subset cost increase of 5% or more. Depending on the preference of a decision maker these could be adjusted. Further increasing the maximum depth of the BST will, however, increase the complexity of determining the marginal cost increase as all possible partitions have to be considered, and the number of partitions increases exponentially with the depth. As a result of increasing the maximum depth, there can be an exponential increase in the number of optimizations that need to be solved. In that case, one could consider pruning the tree after reaching a certain cost, or by only considering food items to be excluded that were in former food baskets of a certain depth. Furthermore, we assumed that accurate prices are readily available. If this is not the case, this can affect the identification of essential foods.

The construction and evaluation of the BST do not need any assumptions on the underlying optimization model. As we wanted a case study relevant to the humanitarian sector, we adopted the underlying optimization model used in the Cost of the Diet software (5, 18). This model can be formulated as an LP, and is easily solvable. Our case study mainly focuses on cost and adherence to nutritional requirements, and depending on the context of the problem, more restrictions can be included to better capture local preferences to encourage the adoption of the presented results by the target population. A limitation of LP is that it cannot capture the intricate and (often) non-linear absorption of nutrients. This could be captured in a non-linear model, which would then serve as the underlying model within the BST.

### 4.2 Insights from the Ebonyi case study

A BST analysis can lead to various valuable insights, which we illustrate with a case study on Ebonyi, Nigeria. The BST can be used to find specific foods that are in general relevant for the construction of low-cost nutritious baskets. For the Nigeria case study, we find that “lamb, liver” is relevant for the affordability of nutritious baskets, as excluding it alone results in a cost increase of more than 10%, and excluding it with other items results in an even larger cost increase. Furthermore, we find that “leaf, amaranth” is relevant. Although excluding “leaf, amaranth” in itself might not impact the cost substantially, “leaf, amaranth” is essential to keep the overall basket more affordable when other nutrient-dense foods are not available. The combined absence of these two items could impose greater challenges in the access to nutritious affordable baskets in this region.

Furthermore, our BST analysis for Nigeria shows that for the general population there are several limiting micronutrients such as calcium, pantothenic acid and vitamin B12. In addition, the (absorbed) iron intake is found to be a key limiting micronutrient for the breastfed child, adolescent female and lactating female, indicating a higher vulnerability of these population groups. In this case, fortification or supplementation with these nutrients could help to lower the cost of nutritious diets.

From the BST analysis, we can see that when animal-source foods such as offal and fish are not available, there is a sharp increase in the cost of a nutritious basket. Animal-source foods are usually rich in nutrients, especially vitamin B12, and even in small amounts they can contribute greatly to a nutrient-rich diet. Excluding both “lamb, liver” and “fish, dried” results in a cost increase of 35% with respect to the minimum-cost food basket, and additionally excluding “fish, mackerel” results in a 56% cost increase. Individuals who normally do not include animal-source foods in their diet might therefore be at greater risk of not being able to meet nutritional requirements.

### 4.3 Future prospects

In the context of the work of the UN World Food Programme, the BST can be used to indicate whether the minimum-cost estimate is robust against possible supply chain disruptions due to, for example, climatic or economic shocks. The approach could be used in the identification of specific food items for nutrition-sensitive commodity lists to be regularly monitored. As extensive data collection is not always viable due to conflict or time constraints, the unavailability or rapid cost increase of these monitored items can be part of early-warning systems.

The BST analysis could also help the design of social assistance programs. In contexts where minimum-cost metrics are used to inform social protection programs (e.g., cash transfers), the BST can be used to determine the degree to which these estimates may be susceptible to specific market shocks and where additional interventions may be needed to fill need gaps. The BST could also be used to support decisions around the modality of social assistance transfers; for example, based on BST results, disruptions in the supply of key food items could see a shift to supplementary or in-kind programs, as nutrient needs may no longer be met with locally available foods.

The BST could also be used to explore specific questions, such as determining whether the nutrient requirements of malnourished children can be met based on local supply. As these children have a greater need for nutrient-dense foods, it is a considerable challenge to meet their nutrient requirements. Providing evidence and identifying a list of key foods may help to propose dedicated interventions.

As the BST shows which (combinations of) food items are the most relevant when they are not included, we suspect that these items will also be the most interesting to investigate within a sensitivity analysis. This raises questions as to how sensitive the minimum-cost estimate is to changes in the price of these items, and whether the reported prices are accurate enough or might need some validation. Furthermore, as there are seasonal fluctuations in prices as well as the availability of food items, the BST can be used to investigate the impact of the unavailability of seasonal items.

At a micro level, the BST can be used to investigate which food items could be introduced in the local market or cultivated as an alternative for other items. For example, one could study whether introducing specific leafy greens can help to reduce the cost of a minimum-cost nutritious basket (22). This approach could be used as part of the FNG analyses to systematically explore inventions to fill nutrient gaps (8).

At a macro level, the results of the BST can help to see whether there is an imbalance in the current levels of import and export. For example, are essential items heavily dependent on import, or are they produced locally? What are the implications of different type of disruptions on the availability of essential items?

The insights into the key limiting nutrients provided by the BST can be used to investigate the possibility of using fortification to reduce the cost of nutritious meals. Fortification adds specific nutrients to certain foods which makes it a cost-effective way to increase access to nutritious foods. For example, in an FNG analysis for Afghanistan it was shown that the cost of a nutritious diet could be reduced by 13 − 22% by replacing the regular wheat flour with fortified wheat flour (23). The fortification consisted of folic acid, vitamin B12, iron and zinc.

### 4.4 Conclusion

To conclude, the construction and evaluation of the BST help to identify indispensable foods of minimum-cost nutrient-adequate food baskets in a concise way. In addition, the BST can detect possible substitute effects between foods and identify key limiting nutrients. As the BST does not need any assumptions on the underlying optimization model, the analysis can be tailored to a specific context. Its results could be used to inform and support decisions related to food and nutrition security, e.g., by the design of social assistance programs or dedicated interventions for malnourished children.

## Data Availability

The original contributions presented in the study are included in the article/Supplementary material, further inquiries can be directed to the corresponding author.

## Footnotes

^1^Note that this can also be a single item.

^2^This is related to the Bell number.

^3^The subset cost increase is 4.4%. As it is below 5%, it is not reported in Table 2.

^4^This is determined by summing 1.55% of “millet” and 4.68% of “sorghum”.

^5^This is determined by summing 35.34% of “lamb, liver” with “fish, dried”, and 4.43% of “leaf, amaranth”.
