# Supplementary material for: A tree-based approach to identify indispensable foods in minimum-cost food baskets
Source: Front Nutr. 2024 Sep 18;11:1425749. doi: 10.3389/fnut.2024.1425749 (PMC11445006; doi:10.3389/fnut.2024.1425749)
Supplement: Supplementary file 1 [file Data_Sheet_1.pdf]

Supplementary Material

1 INPUT DATA CASE STUDY

Table S1 reports the recommended daily intake of these five household members for all considered nutrients $\mathcal{N}$ and it states the maximum food weight w for each individual, both according to the CotD guidelines (Deptford and Hall, 2014). Here, we assume that the breastfed child receives a daily amount of 532 grams of breastmilk (Deptford and Hall, 2014), and we take this into account for the reported nutritional requirements and maximum food weight. Note that depending on the application other nutrients (e.g. fiber, saturated/unsaturated fats) can be added to this list.

In short, the energy intake is based on the estimated average requirement (EAR) of the World Health Organization (WHO) and Food and Agriculture Organization (FAO) (FAO/WHO, 2001), which indicates that for a given individual there is a 50% probability that their energy needs are met. The protein intake is based on data from WHO/FAO (FAO/WHO, 2007), where the intake ensures that for a given individual there is a 95% probability that their protein needs are met. The fat intake is based on recommendations from WHO/FAO(FAO/WHO, 2008), where for a given individual they state a percentage range of energy that should be provided by fat. For adolescent individuals, this is usually between $20 - 35\%$. The table shows the converted intake in grams of fat.

For all other specified nutrients, the lower limit is the recommended nutrient intake (RNI) based on data from FAO/WHO. In general, an RNI of a nutrient indicates a sufficient value to meet the requirements of $97 - 98\%$ of healthy individuals in a specific group. This is determined by the EAR plus two standard deviations. Note that this definition is equivalent to the recommended dietary allowance (RDA). For some nutrients an upper limit (UL) is specified, either obtained from WHO/FAO, Institute of Medicine, or the European Food Safety Authority (EFSA).

Table S2 lists the available food items and the corresponding food groups, obtained for the region Ebonyi, Nigeria. Furthermore, it reports for each household member their portion size p_i of each food item.

Table S1. Daily nutritional requirements for the considered household. The reported ranges for the five household members reflect the minimum and maximum allowance for each nutrient. In case only a lower or an upper limit is applied, this is indicated with $\leq$ or $\geq$, respectively. Only the energy intake is modeled as an exact equality.

	Source	Type	Breastfed child*	School-age child	Adolescent female	Lactating female	Male
Energy (kcal)	(FAO/WHO, 2001)	EAR	561.2	1501	2449	2760	2750
Protein (g)	(FAO/WHO, 2007)	95p	≥ 7.96	≥ 17.84	≥ 46.27	≥ 55.36	≥ 40.40
Fat (g)	(FAO/WHO, 2008)	%E	$9.47 - 19.54$	$41.68 - 58.35$	$68.03 - 95.24$	$61.33 - 107.33$	$61.11 - 106.94$
Calcium (mg)	(FAO/WHO, 2002, 2004)	RNI, UL	$351.04 - 4735.04$	$600 - 3387$	$1300 - 2727$	$1000 - 4985$	$1000 - 2825$
Folic acid (µg DFE)	(FAO/WHO, 2004)	RNI	≥ 104.78	≥ 200	≥ 400	≥ 500	≥ 400
Iron absorbed (mg)	(FAO/WHO, 2004)	RNI	≥ 0.58	≥ 0.63	≥ 3.10	≥ 2.94	≥ 1.37
Iron (mg)	**		≤ 25	≤ 70	≤ 120	≤ 120	≤ 90
Magnesium (mg)	(FAO/WHO, 2004)	RNI	≥ 41.38	≥ 76	≥ 220	≥ 270	≥ 260
Niacin (mg NE)	(European Commission, 2001; FAO/WHO, 2004)	RNI, UL	$3.69 - 241.69$	$8 - 248$	$16 - 541$	$17 - 1696$	$16 - 848$
Pantothenic acid (mg)	(FAO/WHO, 2004)	RNI	≥ 1.04	≥ 3	≥ 5	≥ 7	≥ 5
Vitamin A (µg RAE)	(FAO/WHO, 2004; Institute of Medicine, 2001)	RNI, UL	$134 - 711$	$450 - 1016$	$600 - 2056$	$850 - 5349$	$600 - 3333$
Vitamin B1 (mg)	(FAO/WHO, 2004)	RNI	≥ 0.39	≥ 0.6	≥ 1.1	≥ 1.5	≥ 1.2
Vitamin B2 (mg)	(FAO/WHO, 2004)	RNI	≥ 0.31	≥ 0.6	≥ 1	≥ 1.6	≥ 1.3
Vitamin B6 (mg)	(FAO/WHO, 2004)	RNI	≥ 0.45	≥ 0.6	≥ 1.2	≥ 2	≥ 1.3
Vitamin B12(µg)	(FAO/WHO, 2004)	RNI	≥ 0.38	≥ 1.2	≥ 2.4	≥ 2.8	≥ 2.4
Vitamin C (mg)	(FAO/WHO, 2002, 2004)	RNI, UL	$8.72 - 1606.72$	$30 - 1129$	$40 - 909$	$70 - 1663$	$45 - 942$
Zinc (mg)	(FAO/WHO, 2004)	RNI	≥ 3.46	≥ 4.8	≥ 7.2	≥ 7.2	≥ 7
Maximum food weight (g), w	(Deptford and Hall, 2014)		≤ 700	≤ 1871	≤ 3053	≤ 3441	≤ 3429

* Requirements take daily intake of 532 grams of breastmilk into account.

** Based on conversations with the UN World Food Programme.

Abbreviations: EAR for estimated average requirement, 95p for 95 percentile, %E for a certain percentage of energy intake, RNI for recommended nutrient intake, UL for upper limit.

Table S2. The available food items with corresponding food groups in Ebonyi, Nigeria. For each considered household member their portion size in grams of each food item is reported.

Food item	Food group	Breastfed child	School-age child	Adolescent female	Lactating female	Male
Bambara groundnut, dried, raw	Legumes, nuts and seeds	15	30	45	45	51
Bean, white, dried	Legumes, nuts and seeds	60	120	180	180	204
Beans, green, raw	Vegetable (products)	195	390	585	585	663
Beets, raw	Roots and tubers	285	570	855	855	969
Cabbage, raw	Vegetable (products)	195	390	585	585	663
Carrot, raw	Vegetable (products)	300	600	900	900	1020
Chicken, clean, ready to cook	Meat and offal	45	90	135	135	153
Cocoyam, tuber, raw	Roots and tubers	180	360	540	540	612
Cowpea, black, dried, raw	Legumes, nuts and seeds	60	120	180	180	204
Cowpea, brown, dried, raw	Legumes, nuts and seeds	60	120	180	180	204
Cucumber, raw	Vegetable (products)	195	390	585	585	663
Dikanut, kernel, dried, raw	Legumes, nuts and seeds	15	30	45	45	51
Egg, chicken, raw	Egg (products)	120	240	360	360	408
Eggplant, white, raw	Vegetable (products)	195	390	585	585	663
Fish, cod, atlantic, raw	Fish, seafood (products)	75	150	225	225	255
Fish, dried, CotD	Fish, seafood (products)	75	150	225	225	255
Fish, mackerel, raw	Fish, seafood (products)	75	150	225	225	255
Fish, tilapia, raw	Fish, seafood (products)	75	150	225	225	255
Goat, feet	Meat and offal	45	90	135	135	153
Goat, meat, raw	Meat and offal	45	90	135	135	153
Groundnut, shelled, dried, raw	Legumes, nuts and seeds	15	30	45	45	51
Guava, fruit	Fruit (products)	165	330	495	495	561
Lamb, liver, raw	Meat and offal	75	150	225	225	255
Leaf, amaranth, raw	Vegetable (products)	285	570	855	855	969
Leaf, eggplant, raw	Vegetable (products)	285	570	855	855	969
Leaf, roselle, raw	Vegetable (products)	285	570	855	855	969
Macaroni, dried	Grains/grain-based	180	360	540	540	612
Maize, white, whole kernel, dried, raw	Grains/grain-based	195	390	585	585	663
Maize, yellow, whole kernel, dried, raw	Grains/grain-based	195	390	585	585	663
Melon, seeds, slightly salted, raw	Legumes, nuts and seeds	30	60	90	90	102
Milk, powder, fortified	Milk and milk products	39	78	117	117	132.6
Millet, pearl, whole grain, raw	Grains/grain-based	165	330	495	495	561
Mushroom, CotD	Vegetable (products)	195	390	585	585	663
Noodle, dried	Grains/grain-based	195	390	585	585	663
Oats	Grains/grain-based	195	390	585	585	663
Oil, groundnut	Oils and fats	30	60	90	90	102
Oil, palm, red	Oils and fats	30	60	90	90	102
Okra, raw	Vegetable (products)	195	390	585	585	663
Onion, red	Vegetable (products)	195	390	585	585	663
Palm nuts, pulp	Fruit (products)	165	330	495	495	561
Peanut, with shell	Legumes, nuts and seeds	15	30	45	45	51
Peas, raw	Vegetable (products)	195	390	585	585	663
Pepper, sweet, red, raw	Vegetable (products)	285	570	855	855	969
Pineapple, pulp	Fruit (products)	165	330	495	495	561
Plantain, ripe, raw	Vegetable (products)	195	390	585	585	663
Potato, raw	Roots and tubers	180	360	540	540	612
Pumpkin, squash, raw	Vegetable (products)	300	600	900	900	1020
Rice, white, long grain, parboiled, unenriched, dry	Grains/grain-based	180	360	540	540	612
Rice, white, raw	Grains/grain-based	180	360	540	540	612
Sesame, seeds, whole, dried, raw	Legumes, nuts and seeds	30	60	90	90	102
Sheep, tripe	Meat and offal	75	150	225	225	255
Shrimp, dried	Fish, seafood (products)	90	180	270	270	306
Sorghum, whole grain, raw	Grains/grain-based	180	360	540	540	612
Soybean, dried, raw	Legumes, nuts and seeds	60	120	180	180	204
Spaghetti, dry, unenriched	Grains/grain-based	180	360	540	540	612
Sweet potato, pale yellow, raw	Roots and tubers	180	360	540	540	612
Tapioca, pearl, dry	Grains/grain-based	180	360	540	540	612
Tomato paste, concentrated	Herbs, spices and condiments	3	6	9	9	10.2
Tomato, red, ripe, raw	Fruit (products)	90	180	270	270	306
Tomato, sundried	Fruit (products)	90	180	270	270	306
Watermelon, fruit	Fruit (products)	90	180	270	270	306
Wheat, whole grain, raw	Grains/grain-based	165	330	495	495	561

2 NUTRITIONAL CONTENT MINIMUM-COST FOOD BASKET

Table S3 reports the daily nutritional composition of the minimum-cost food basket for each household member. Furthermore, it reports the daily cost for each individual. Note that we do not report the energy intake, as this is assumed to be exact.

Table S3. Daily nutritional composition of the minimum-cost food basket for each household member. In addition, the daily cost for each individual is reported. Underlined indicates the reported value is at the lower limit, and **bold** indicates at the upper limit.

	Breastfed child	School-age child	Adolescent female	Lactating female	Male
Protein (g)	18.33	52.41	90.95	107.09	88.20
Fat (g)	9.47	41.68	68.03	61.33	61.11
Calcium (mg)	351.04	600	1770.33	1000	1000
Folic acid (μg DFE)	115.85	272.64	636.29	626.96	447.46
Iron absorbed (mg)	0.65	1.18	3.10	3.73	2.50
Iron (mg)	13.02	23.00	55.09	46.21	49.40
Magnesium (mg)	392.64	1167.01	1788.50	2267.78	1827.57
Niacin (mg NE)	5.92	21.85	36.96	45.39	32.62
Pantothenic acid (mg)	1.04	3	5	7	5
Vitamin A (μg RAE)	225.44	450	**2056**	4667.16	668.61
Vitamin B1 (mg)	0.57	1.74	2.79	3.25	2.93
Vitamin B2 (mg)	0.59	1.12	3.09	4.35	2.19
Vitamin B6 (mg)	0.85	1.52	3.45	3.16	3.61
Vitamin B12(μg)	0.38	2.38	18.57	75.18	2.40
Vitamin C (mg)	38.01	58.89	192.69	98.97	99.18
Zinc (mg)	3.91	8.43	16.53	18.39	17.38
Cost (NGN)	66.63	186.68	318.57	353.53	324.38

3 NUTRITIONAL CONTENT OF EXCLUSION SETS FOR ADOLESCENT FEMALE

Table 3 reports the daily nutritional composition corresponding to the food baskets of the exclusion sets of Table 2 for the adolescent female. The nutritional composition for the category ‘zero items excluded’ matches with the composition of the adolescent female as presented in Table 2. Note that we do not report the energy intake, as this is assumed to be exact.

Table S4. Daily nutritional composition and cost of the exclusion sets of Table 2 for the adolescent female. Underlined indicates the reported value is at the lower limit, and **bold** indicates at the upper limit.

Zero items excluded					Cost (NGN)	Protein (g)	Fat (g)	Ca (mg)	FA (µg DFE)	Iron Abs (mg)	Iron (mg)	Mg (mg)	Niacin (mg NE)	PA (mg)	Vit A (µg RAE)	Vit B1 (mg)	Vit B2 (mg)	Vit B6 (mg)	Vit B12 (µg)	Vit C (mg)	Zinc (mg)
—					318.57	90.95	68.03	1770.33	636.29	3.10	55.09	1788.50	36.96	5	**2056**	2.79	3.09	3.45	18.57	192.69	16.53
**One item excluded**																					
Lamb, liver					354.27	110.62	68.03	2541.85	699.91	3.10	59.48	1927.62	41.70	5	1369.09	2.70	2.88	3.45	2.4	254.69	16.55
**Two items excluded**																					
Sorghum	Lamb, liver				367.52	94.27	68.03	1455.03	481.49	3.10	59.23	941.21	36.98	5	700.56	2.44	2.62	4.86	2.4	133.02	19.35
Leaf, amaranth	Sorghum				354.46	70.88	75.26	1300	400	3.10	48.95	831.38	20.24	5	**2056**	2.23	2.66	4.41	34.00	75.58	22.42
Leaf, amaranth	Sesame seeds				374.55	140.53	68.03	1300	1493.35	3.10	50.92	1473.51	21.45	5	**2056**	3.52	3.12	4.81	28.77	150.87	24.25
Leaf, amaranth	Lamb, liver				415.57	91.12	68.03	1896.42	549.45	3.10	59.06	935.60	28.89	5	781.03	2.55	2.82	5.29	2.4	210.58	23.20
Millet	Sorghum				364.74	141.29	68.03	1675.35	2231.47	3.10	53.06	1452.56	40.08	5	**2056**	4.35	2.74	3.27	22.22	159.55	24.22
Lamb, liver	Fish, dried				430.44	104.26	68.03	2073.72	633.19	3.10	58.99	1724.97	37.75	5	1254.03	2.71	2.84	3.87	2.4	226.31	16.33
**Three items excluded**																					
Leaf, amaranth	Lamb, liver	Leaf, eggplant			472.47	95.22	**95.24**	1300	400	3.10	46.55	823.67	18.20	5.17	600	2.19	2.20	4.25	3.28	40	22.76
Leaf, amaranth	Lamb, liver	Fish, dried			469.70	89.72	68.03	1300	477.19	3.10	51.88	1015.57	20.48	5	765.14	2.43	2.73	4.72	2.4	148.83	20.63
Leaf, amaranth	Sesame seeds	Leaf, eggplant			398.85	186.10	91.52	1300	2044.18	3.10	47.04	1514.00	20.39	5	**2056**	4.01	2.84	4.88	37.66	40	27.99
Millet	Sorghum	Lamb, liver			397.13	151.07	68.03	2544.69	2039.93	3.10	58.90	1520.00	43.28	5	1256.92	3.90	2.54	3.40	2.4	237.46	22.95
Leaf, amaranth	Millet	Lamb, liver			473.38	167.05	68.03	**2727**	1836.45	3.10	56.16	1837.34	26.87	5	1642.39	3.80	3.59	4.88	2.4	417.87	23.75
Lamb, liver	Fish, dried	Fish, mackerel			498.58	91.73	68.03	1300	481.55	3.96	60.05	820.85	16	6.50	979.65	2.00	3.39	4.82	2.4	103.02	21.15
**Four items excluded**																					
Leaf, amaranth	Millet	Lamb, liver	Fish, dried		532.97	194.31	68.03	1839.46	2721.83	3.10	53.31	1528.18	22.50	5	948.08	4.74	2.82	4.70	2.4	212.45	29.19
Leaf, amaranth	Lamb, liver	Oil, palm	Leaf, eggplant		477.49	89.00	93.21	1300	400	3.10	48.14	844.23	16.66	5.14	600	2.26	2.20	4.40	2.4	40	23.19
Leaf, amaranth	Millet	Sorghum	Lamb, liver		478.43	201.63	68.03	2514.62	2775.29	3.10	58.66	1614.81	29.06	5	1158.90	4.78	3.12	5.14	2.4	312.15	30.22
Leaf, amaranth	Lamb, liver	Sesame seeds	Leaf, eggplant		511.88	165.49	68.03	1300	648.09	3.10	41.47	812.46	35.72	5.41	600	2.18	2.22	4.69	10.01	40	21.60
Leaf, amaranth	Millet	Lamb, liver	Leaf, eggplant		546.93	159.53	74.36	1300	2389.76	3.10	43.42	1385.78	19.56	6.55	600	4.37	2.11	2.94	2.4	40	30.14
Millet	Sorghum	Lamb, liver	Fish, dried		476.41	156.74	68.03	2150.02	2252.20	3.10	58.43	1565.28	41.58	5	1158.56	4.36	2.50	3.57	2.4	212.63	23.89
Leaf, amaranth	Sesame seeds	Soybean	Leaf, eggplant		436.54	167.41	68.03	1300	400	3.10	35.17	1001.76	60.44	5	1976.77	2.38	2.58	4.24	46.97	40	19.33
Leaf, amaranth	Lamb, liver	Leaf, eggplant	Egg, chicken		636.88	213.90	68.03	**2727**	400	3.10	41.31	1012.63	66.41	5.46	600	2.17	1.72	4.63	18.14	40	23.77
Lamb, liver	Fish, dried	Fish, mackerel	Egg, chicken		661.54	107.87	68.03	1587.42	570.01	3.10	54.40	1536.69	36.24	5	950.06	2.57	2.44	3.84	2.4	169.49	16.77
**Five items excluded**																					
Leaf, amaranth	Sorghum	Cucumber	Rice, white	Mushroom	384.70	75.86	68.03	1300	573.03	3.10	49.33	887.26	19.16	5	**2056**	2.87	2.76	4.52	31.71	141.84	22.87
Leaf, amaranth	Millet	Sorghum	Lamb, liver	Fish, dried	532.97	194.31	68.03	1839.46	2721.83	3.10	53.31	1528.18	22.50	5	948.08	4.74	2.82	4.70	2.4	212.45	29.19
Leaf, amaranth	Millet	Sorghum	Sesame seeds	Soybean	440.31	152.69	68.03	1300	2682.40	3.10	48.58	1373.46	33.52	5	**2056**	4.91	2.98	3.35	22.53	209.78	26.41
Leaf, amaranth	Millet	Lamb, liver	Oil, palm	Leaf, eggplant	554.99	162.36	69.85	1300	2446.52	3.10	43.99	1408.56	20.20	6.58	600	4.46	2.11	3.05	2.4	40	30.47
Leaf, amaranth	Lamb, liver	Sesame seeds	Oil, palm	Leaf, eggplant	527.23	168.22	68.03	1300	850.08	3.10	43.11	894.29	32.59	5.48	600	2.45	2.26	4.87	8.67	40	22.71
Leaf, amaranth	Lamb, liver	Sesame seeds	Leaf, eggplant	Egg, chicken	689.90	272.43	68.03	**2727**	910.77	3.10	38.20	1163.94	75.18	6.58	600	2.60	1.95	4.55	20.77	47.51	23.49
Leaf, amaranth	Millet	Sesame seeds	Soybean	Cowpea	540.75	105.29	68.03	1300	1478.73	3.10	50.93	1544.38	24.78	5	**2056**	3.40	2.99	2.70	20.46	246.00	19.23
Sorghum	Lamb, liver	Rice, white	Mushroom	Cucumber	390.62	106.95	68.03	1888.74	581.96	3.10	59.05	1127.08	31.71	5	941.49	3.02	2.53	3.96	2.4	175.54	20.74
Leaf, amaranth	Millet	Sorghum	Soybean	Cowpea	511.38	102.61	**95.24**	1938.64	1225.06	3.10	47.62	1202.39	20.05	5	**2056**	3.48	2.09	3.12	37.10	40	28.52
Millet	Sorghum	Lamb, liver	Fish, dried	Fish, mackerel	555.26	166.00	68.03	1300	2509.19	3.30	46.44	1417.09	22.51	6.97	619.03	4.47	2.44	3.04	2.4	40	29.45
Leaf, amaranth	Millet	Lamb, liver	Leaf, eggplant	Egg, chicken	694.21	238.20	68.03	**2727**	1575.11	3.10	43.43	1329.57	62.38	5	600	3.39	1.31	3.46	16.29	40	28.62
Leaf, amaranth	Lamb, liver	Fish, dried	Leaf, eggplant	Egg, chicken	729.96	101.68	75.54	1300	400	3.10	59.11	1179.76	40.23	8.10	600	3.20	2.45	4.33	2.4	109.20	21.65
Leaf, amaranth	Lamb, liver	Fish, dried	Sesame seeds	Leaf, eggplant	629.35	192.36	**95.24**	1300	2175.08	3.10	46.62	1354.61	16	6.26	600	4.01	2.63	4.55	2.4	40	27.69
Leaf, amaranth	Lamb, liver	Oil, palm	Leaf, eggplant	Carrot	571.83	89.26	86.21	1300	400	3.10	50.60	867.50	18.84	5.52	600	2.36	2.06	4.54	2.4	40	23.32
Leaf, amaranth	Sesame seeds	Soybean	Leaf, eggplant	Fish, dried	896.79	139.61	68.03	1300	2760.64	3.10	53.84	1406.83	32.02	5	**2056**	5.01	3.48	3.58	21.58	170.78	26.65

Abbreviations: Ca for calcium, FA for folic acid, Iron abs for iron absorbed, Mg for magnesium, PA for pantothenic acid, and Vit for vitamin.

REFERENCES

[Dataset] Deptford, A. and Hall, A. (2014). Cost of the Diet. A practitioner's guide. Version 2.

[Dataset] European Commission (2001). Tolerable upper intake levels for vitamins and minerals. `http://ec.europa.eu/food/fs/sc/scf/out80_en.html`. Accessed: 2014-06-18

FAO/WHO (2001). *Human energy requirements* (WHO, Geneva, Switzerland)

FAO/WHO (2002). *Human vitamin and mineral requirements* (WHO, Geneva, Switzerland)

FAO/WHO (2004). *Vitamin and mineral requirements in human nutrition* (WHO, Geneva, Switzerland)

FAO/WHO (2007). *Protein and amino acid requirements in human nutrition* (WHO, Geneva, Switzerland)

FAO/WHO (2008). *Fats and fatty acids in human nutrition* (WHO, Geneva, Switzerland)

Institute of Medicine (2001). *Dietary reference intakes for vitamin A, vitamin K, arsenic, boron, chromium, copper, iodine, iron, manganese, molybdenum, nickel, silicon, vanadium, and zinc* (National Academy Press Washington, DC, USA)
